# Biosensors Integration in Blood–Brain Barrier-on-a-Chip: Emerging Platform for Monitoring Neurodegenerative Diseases

**DOI:** 10.1021/acssensors.2c00333

**Published:** 2022-05-13

**Authors:** Mònica Mir, Sujey Palma-Florez, Anna Lagunas, Maria José López-Martínez, Josep Samitier

**Affiliations:** † Biomedical Research Networking Center in Bioengineering, Biomaterials, and Nanomedicine (CIBER-BBN) Monforte de Lemos 3-5, Pabellón 11, 28029 Madrid, Spain; ‡ Nanobioengineering Group, Institute for Bioengineering of Catalonia (IBEC), Barcelona Institute of Science and Technology (BIST), 12 Baldiri Reixac 15-21, Barcelona 08028, Spain; § Department of Electronics and Biomedical Engineering, University of Barcelona, Martí i Franquès 1, 08028 Barcelona, Spain

**Keywords:** organ-on-a-chip (OoC), biosensors, blood−brain barrier (BBB), transepithelial/transendothelial electrical resistance (TEER), neurodegenerative diseases (NDDs)

## Abstract

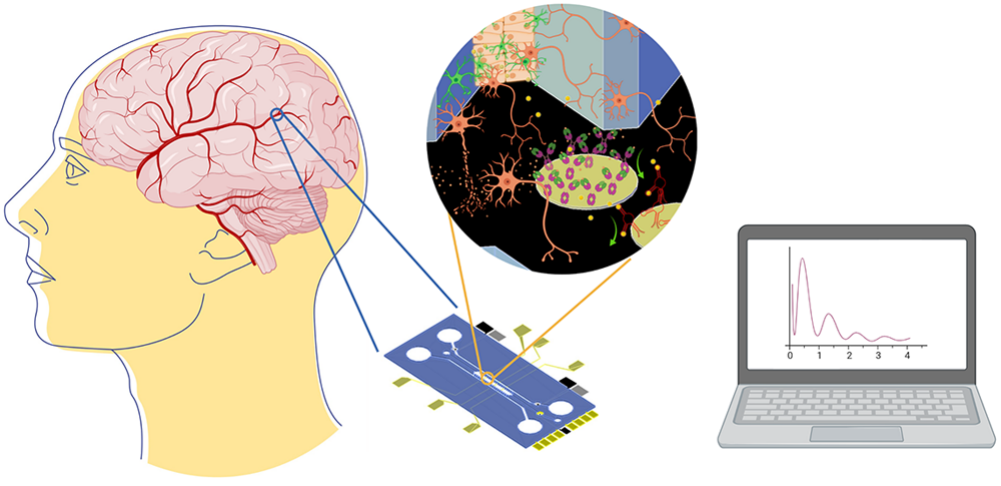

Over the most recent decades, the development of new biological platforms to study disease progression and drug efficacy has been of great interest due to the high increase in the rate of neurodegenerative diseases (NDDs). Therefore, blood–brain barrier (BBB) as an organ-on-a-chip (OoC) platform to mimic brain-barrier performance could offer a deeper understanding of NDDs as well as a very valuable tool for drug permeability testing for new treatments. A very attractive improvement of BBB-oC technology is the integration of detection systems to provide continuous monitoring of biomarkers in real time and a fully automated analysis of drug permeably, rendering more efficient platforms for commercialization. In this Perspective, an overview of the main BBB-oC configurations is introduced and a critical vision of the BBB-oC platforms integrating electronic read out systems is detailed, indicating the strengths and weaknesses of current devices, proposing the great potential for biosensors integration in BBB-oC. In this direction, we name potential biomarkers to monitor the evolution of NDDs related to the BBB and/or drug cytotoxicity using biosensor technology in BBB-oC.

In the most recent decades, age-dependent diseases such as neurodegenerative diseases (NDDs) have become more prevalent, partly because life expectancy has increased. Unfortunately, the efficacy of pharmacological treatment of these NDDs has very high preclinical and clinical failure rates with the worst outcomes observed in Alzheimer’s disease (AD), Parkinson’s disease (PD), amyotrophic lateral sclerosis (ALS), and neuromuscular disorders. In the case of AD, it is estimated that developing a disease-modifying treatment could take about 13 years and cost more than $5.5 billion. But if even a regulated success is achieved, the prevalence of the disease could be halved in just 5 years by the year 2050.^1,2^ Animal studies remain the gold standard for preclinical validation of drugs in pharmaceutical development. However, their results for predicting success in NDDs clinical assays have been disappointing as the accuracy and reproducibility of the results obtained are impaired due to species differences between animal and human systems. Moreover, the use of animal models is expensive, time-consuming, and subjected to ethical constraints. Therefore, efforts are focused on the development of in vitro platforms that reproduce both the physiological and pathological scenarios. Organs-on-a-chip (OoC) are microengineered biomimetic systems able to recapitulate key functions of living organs. They are microfluidic platforms created with manufacturing methods, used in microchip technology, which contains a cell culture in perfused chambers.^3^ By integrating living cells cultures in these microfluidic platforms, the most relevant biological and mechanical properties of minimal organic functional units can be reproduced. OoC in vitro models are an interesting alternative due to their ability to reliably reproduce biological characteristics of in vivo physiological and pathological conditions with human based cells for the study of disease progression, drug testing, and drug permeability, among others.

The study of blood–brain barrier (BBB) physiology raises special interest since it is one of the most extensive and restrictive developed barriers in the central nervous system, which acts as a natural guard protecting the brain from the entrance of neurotoxic agents, drugs, invading pathogens, and circulating blood cells.^4−7^ Therefore, BBB is key to the study of brain-directed drugs to reduce drug development failure and to study BBB dysfunction that is linked to many NDDs, making the BBB an interesting in vitro model to be developed in an OoC. In the reported BBB-oCs, most of the works monitor the correct evolution of the BBB through microscopy images, while just a few examples are describing other techniques, such as electrodes integration in the chip for the automatable read out. Transendothelial electrical resistance (TEER) is the only integrated detection technique in BBB-oC used to study the permeability and cell behavior of the BBB endothelial cell (EC) layer.^33^ However, TEER offers very limited information and its lack of specificity makes it not entirely conclusive and highly dependent on the environment and experimental settings displaying highly variable results.

However, there are other technologies that can be integrated into OoC that can offer many advantages and open new applications, such as biosensors. They are often applied in medical diagnosis and other areas and are capable of detecting almost any type of analyte selectively and sensitively. The integration of biosensors may bring many advantages on BBB-oC for reaching an automatized monitoring of a wide range of analytes and biomarkers as a throughput device for a personalized study of the diseases or drug testing in NDDs.

In this Perspective, the BBB-oC configurations found in literature are summarized and TEER sensing and influencing factors are presented by providing solutions to overcome them. Moreover, described here is a prospective view of new perspectives of this technology by integrating biosensors into BBB-oC to achieve a high-throughput system that could reach the market for personalized medicine and drug detection in NND. In this direction, the most relevant biomarkers related to BBB dysfunction in NDDs are described, as well as the possibilities offered by different biosensors technologies for their specific detection.

## BBB-oC Platforms for Physiological and Pathological Mimicking of the Brain

### Cell Types Involved in Blood–Brain Barrier-on-a-Chip

BBB is a complex tubular branched network composed of an EC barrier, linked by TJs and surrounded in the parenchymal by pericytes and astrocyte cells. This physical barrier keeps apart blood from neural tissue regulating the molecular transport and acts as a metabolic and immunological barrier.^8^ BBB-oC technology offers the ability to tune geometry, mechanical, and biochemical factors to mimic the human environment in vivo.

There are some examples of BBB-oC found in literature that show the capability to mimic in vivo environment better than the standard Transwell assays, a static membrane-based old technology that is the gold standard of in vitro model for permeability studies of biological barriers. The introduction of microfabricated platforms combined with dynamic flow offers a more realistic design, in which cellular shear stress can be applied on the EC, mimicking the mechanical stimulation produced by the blood flow in vivo, and allowing 3D cell culture through embedding hydrogel with cells inside the microchannel, which render models closer to in vivo conditions, increasing the biological relevance. However, cell characterization through imaging is not completely adapted for 3D structures inspection in the chip since 3D configuration introduces some technical issues.

Regarding cell type, first BBB-oC models included only one type of cultured cells: ECs mainly from mouse and mice origin. However, several studies reported that astrocytes have a key role in the BBB function because they modulate the protein expression, endothelium differentiation, and the formation and maintaining of the TJs.^9,10^ They also showed a protector/clearance performance over disrupting substances such as histamine.^11^ In the same way, other authors have included more than two cellular types to mimic the BBB in a more accurate manner, employing ECs, pericytes, astrocytes, neurons, and microglia. Remarkably, the inclusion of pericytes in the system displayed a higher barrier restriction and low permeability of [^14^C]-mannitol and [^14^C]-urea in the BBB-oC. To include pathological conditions, some authors cocultured ECs and tumoral cells such as glioblastoma (U87). They tested the coculture of these two cell types to develop a tool for future high-throughput screening of different antitumor drugs and evaluate their efficiency to crossing the BBB. To go toward a more realistic physiological barrier, recent BBB-oC models included human primary bone marrow-derived mesenchymal stem cells (BM-MSCs)^12^ or even brain microvascular endothelial cells (BMECs) from human induced pluripotent stem cells (hiPSCs). More recently, some authors created a 3D self-organized microvascular model of the human BBB with hiPSC-ECs and primary pericytes and astrocytes, even hiPSC-derived BBB microvessels, validating barrier function and EC behavior. The use of hiPSCs brings important new future applications for OoC platforms in personalized medicine, as they can be obtained directly from the patient allowing drug testing and disease monitoring for each patient. Recent progress has been performed to generate AD, PD, and Huntington’s disease models from patient-derived iPSCs.^13^ Nevertheless, although paving the way to personalized BBB models, the use of hiPSCs is subjected to the efficacy of the differentiation process and there is a need for more standardized protocols.^14^

### Chip Designs for Blood–Brain Barrier-on-a-Chip

**Figure 1:**
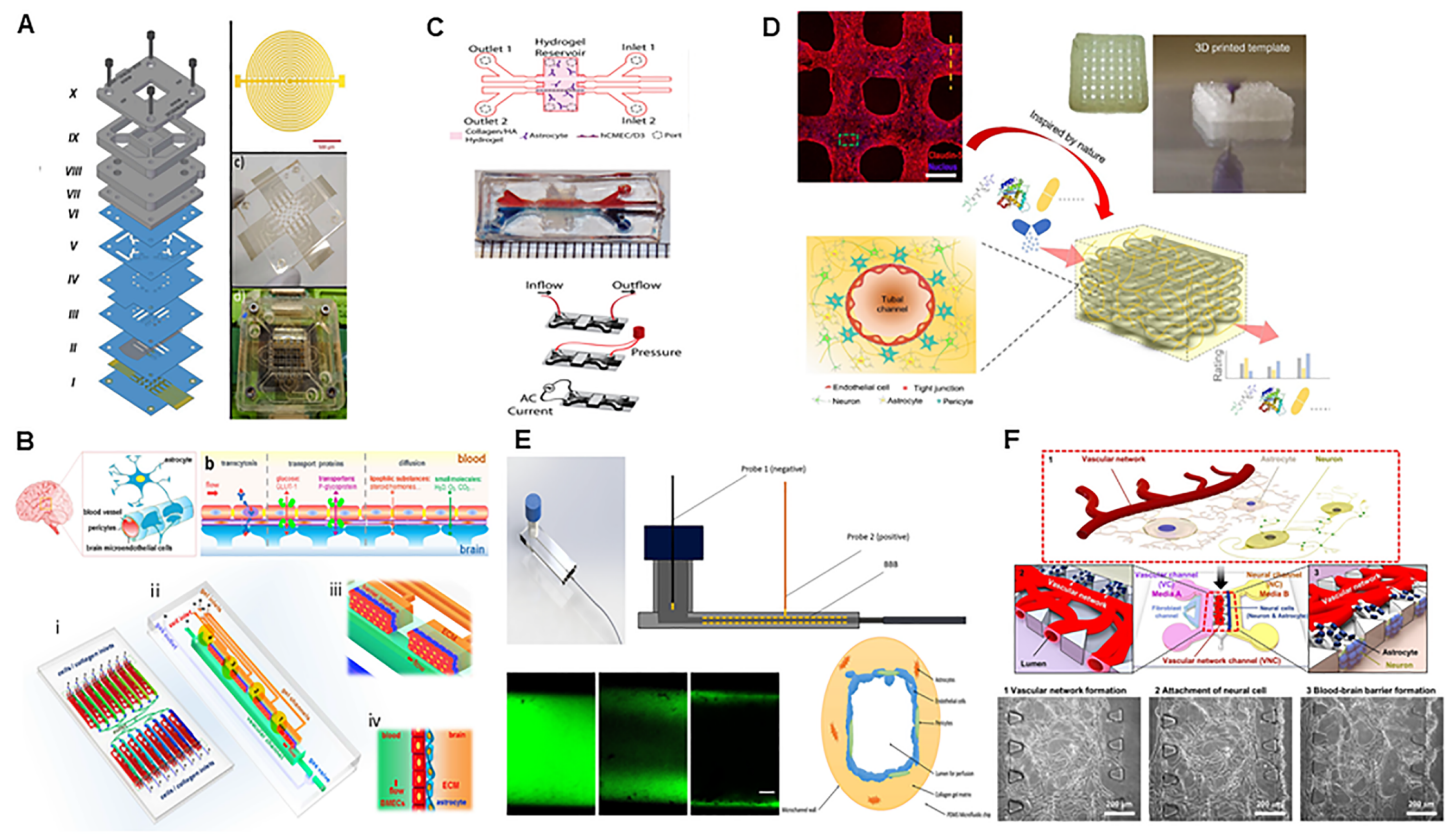
Pictures showing different approaches for BBB-oC configuration. (A) PMMA layers in stack conformation with TEER system included. Reproduced with permission from (19). Copyright 2021 Elsevier. (B) Flank configuration consisting of two layers of PDMS using a collagen gel to mimic the natural extracellular matrix in brain. Reproduced with permission from ref (20). Copyright 2016 The Authors under Creative Commons Attribution 4.0 International License, published by Springer Nature. (C) PDMS layers in flank position and hydrogel consisting in collagen, matrigel and hyaluronic acid and its TEER system. Reproduced with permission from ref (22). Copyright 2017 Elsevier. (D) Polycaprolactone/poly(d,l-lactide-*co*-glycolide) (PCL/PLGA) microfluidic tubular configuration was made by freeze-coating a 3D-printed sacrificial template. Reproduced with permission from ref (24). Copyright 2020 Elsevier. (E) Tubular structure microchannel via viscous finger patterning technique using type I collagen hydrogel and its TEER system included, Reproduced with permission from ref (25). Copyright 2020 Wiley. (F) PDMS devices used to perform a vasculogenesis model. Reprinted with permission from ref (27). Copyright 2017 The Authors under Creative Commons Attribution 4.0 International License, published by Springer Nature.

To faithfully reproduce the physiological scenario, BBB-oC designs are presented in the literature mainly in three different configurations: stack, flank, and tubular. In the stack or vertical configuration, two channels are piled up containing a membrane between them that separates the endothelial and the neuronal culture. This fabrication method is more complex because it requires the assembling of the chip components at micrometric precision but allows the use of different types of membranes embedded between the two channels. This membrane separated EC culture in one channel from neuronal cells in the other channel. As mentioned above, the intercommunication of neuronal and EC is necessary for proper TJs development. Most of the published works used a commercial membrane, but other authors fabricated their own membranes with a desirable material, thickness, and pore size.^15−18^ However, the use of a very thick membrane could discourage cell–cell interaction and hinder the observation of cell cultures on the top of the membrane, limited by the optical working distance of the microscope objective and the transparency of the membrane. Remarkably, some authors have incorporated TEER systems in this kind of configuration, using two PDMS layers enclosing two channels and separated by a porous polycarbonate (PC) membrane with 0.4 mm pores and 10 μm thickness. PDMS layers were placed between two glass slides, and Ag was sputtered for electrodes fabrication. More recently, integrated electrodes and multifrequency TEER with machine learning algorithms have been incorporated in multilayered microfluidic platform using PMMA (Figure 1A).^19^

To facilitate optical inspection, other authors preferred the flanked or horizontal distribution where two or three channels, separated by pillars, are patterned in the same layer. Pillars are usually distributed at short distance along the middle channel to set a barrier between the cell cultures. In evolved systems, 3D hydrogel with cultured cells is used to substitute the commercial membrane (Figure 1B,C).^20,22,23^ This fabrication approach is easier to industrialize than other configurations, which are currently commercialized by companies such as MIMETAS.^21^ Usually, a positive electrode at the input and a negative electrode at the output are used for TEER measurements in this distribution (Figure 1C). The third arrangement in BBC-oC is based on tubular structures to mimic the cylindrical-like brain capillaries geometry based on the formation of hollow fibers as scaffolds that allows the tubular shape. Different fabrication methods have been used, but most of them rely on a sacrificial layer that defines the cylinder (Figure 1D,E).^24,25^ Moreover, in the line of a hollow build up, PDMS is used to hold a wire where researchers gelled Type I collagen and agarose around it. Then, they remove this wire, creating a bare channel for better mimicking of human brain venules.^26^ In a different way, two-photon lithography is employed to obtain microtubes and reproduce a biomimetic/biohybrid BBB model at 1:1 scale.^15^ For the TEER determination, electrodes are connected into the lumen and outside of the BBB of the chip (Figure 1E).^25^ Another interesting approach for 3D BBB fabrication on a chip is the technique mimicking the natural formation and maintenance of our vasculature, angiogenesis (Figure 1F).^27^ Some examples are the works carried out in the Kamm laboratory, among others, where human umbilical vein endothelial cells (HUVECs) were used for new EC to sprout and split perpendicularly to the initial EC channel for the growing of new secondary vessels with morphology similar as natural ones.^23,25,27^

Currently, some examples of BBB-oC are commercially available and in some cases including integrated TEER read out. AIM biotech launched a platform that allows 40 simultaneous experiments on a single plate. They provide protocols for creating 3D cultures seeding different types of cells in a set of three interconnected channels. Elveflow offers a complete kit of a microfluidic platform and flow controllers for OoC experiments, and Alveolix displays a chip to model a wide range of tissue barriers that allows barrier integrity measurements. Moreover, Emulate Co. provides from shell a BBB-oC including five human cell types: neurons, astrocytes, pericytes, microglia, and brain microvascular endothelial cells to mimic the morphological and functional characteristics of cortical brain tissue. MIMETAS Co. developed a multi-BBB-oC in a plate format, named Organoplate. This platform includes high-throughput TEER measurements for their commercial chip. This system allows one to measure up to 40 samples at once in less than a minute, providing an efficient platform to evaluate the barrier permeability.^21^

## TEER Sensing in BBB-oC

A very attractive element in OoC is the integration of detection systems, as it will allow the continuous monitoring of the cells in the chip in real time. As well, the integration of sensors in OoC offers fully automated analysis, increasing their commercial interest.

One of the most important factors in TEER measurements is correlated with the electrodes position. To perform precise TEER measurements and minimize noise, the electrodes should be located close to the cell monolayer in a fixed distance and position. The measurement of in vivo BBB permeability in humans is not straightforward, and this value changes from young to old and from healthy to sick. Several authors have obtained high TEER values in their BBB-oC designs, closer to in vivo (1500–8000 Ω·cm^2^).^32,33^ However, most of them present designs of electrodes located far from the cell monolayer, which means a high-resistance contribution from the solution. Therefore, other authors proposed the used of fixed electrodes as Ag/AgCl thin-film electrode in the top and bottom of the membrane and using a four-probe-method measurement system, which is based on the use of two electrodes to transport the current and two others to detect the voltage.^37^ Some disadvantages of this strategy are the specialized cleanroom requirement for the electrode’s fabrication as well as the lack of visualization of the cell barrier by microscopy due the electrodes position over the membrane. To overcome these drawbacks, electrodes wires inserted into guiding channels at the top and bottom layers allow visualization of the cell monolayer.

Another relevant factor for a precise TEER determination is the uniformity of the current density through the cell culture. For this purpose, properties such as the ratio between the electrode and membrane area and the shape of the electrode play a key role in reaching a homogeneous current density^28,34^ because small electrodes against a large membrane cannot produce enough current across it; leading to an overestimation of TEER. A circular electrode design located in the top and bottom of the cylindrical vertical BBB-oC allowed a relatively uniform electrical current density across the membrane.^38^

**Figure 2:**
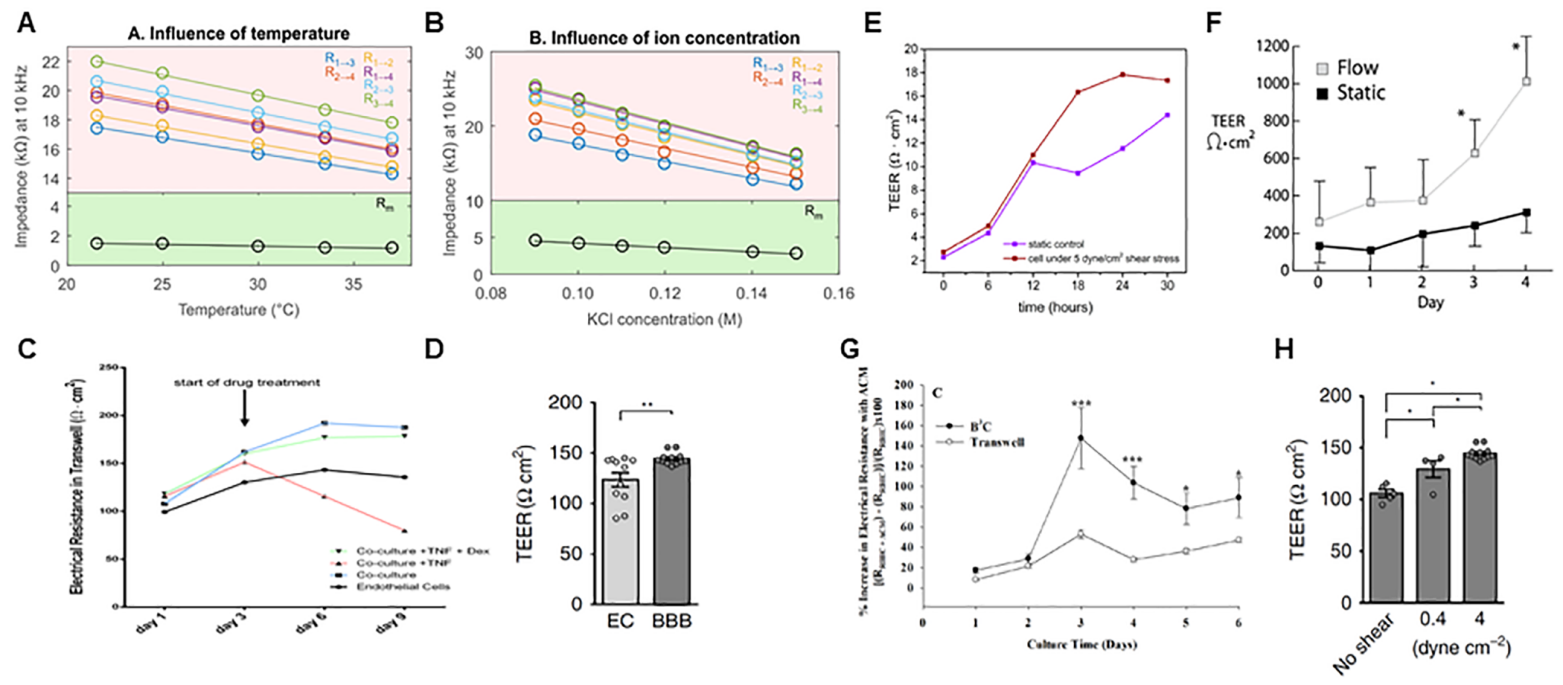
Elements that determine TEER values: (A) temperature and (B) ion concentration effect over impedance recorded at 10 kHz. Reproduced with permission from ref (44). Copyright 2016 Elsevier. (C and D) Increased TEER values of ECs cocultured with other neurovascular cells compared to alone. Panel C reproduced with permission from ref (25). Copyright 2020 Wiley. (E–H) Increasing of TEER values by shear stress in a dynamic system over a static control. Panels D and H reproduced with permission from ref (42). Copyright 2020 The Authors under Creative Commons Attribution 4.0 International License, published by Springer Nature. Panel E reproduced with permission from ref (45). Copyright 2021 The Authors under Creative Commons Attribution 4.0 International License, published by MDPI. Panel F reproduced with permission from ref (22). Copyright 2017 Elsevier. Panel G reproduced with permission from ref (40). Copyright 2015 The Authors under Creative Commons Attribution 4.0 International License, published by PLOS.

Moreover, the TEER measurement depends on the electrode material. It has been observed the influence between the uses of gold (Au) or indium tin oxide (ITO) electrodes in the TEER values by impedance spectroscopy. Other extensively employed material for electrodes in BBB-oC is Ag/AgCl due to their nonpolarizable properties and has a lower cost.^12,16,21,24,38−42^ Unfortunately, solid state Ag/AgCl electrodes can lead to surface degradation with subsequent signal drift and cytotoxicity effects.^30^ To avoid these problems from Ag/AgCl electrodes, other authors used different materials such as Pt.^35^ However, it is important to note that Pt, in contrast to Ag/AgCl, presents a significant interfacial resistance of electrodes medium that may bring signal instability, although it can be eliminated by a four-probes system.^34^ Temperature and the ionic composition of the cell culture medium are also other variables to be considered in all of the electrochemical measurements such as TEER.^30,43^ Previous results revealed that the resistance decreased with increasing temperature and ionic concentration (Figure 2A,B), showing a decrease in sensitivity of 11-fold for temperature and 5-fold for ion concentration ranges.^44^ To minimize inaccuracies in TEER measurements, room temperature needs to be stable and changing the medium before recording each of the impedance spectra, using always the same medium in the measurement.

As well as the TEER measurement setup, the BBB phenotype used in the barrier is an important factor in TEER results as it can be critically affected by factors such as the type of cells included in the barrier, cell differentiation factors, and the shear stress.^46−50^ So far most BBB-oC works showed increasing TEER values when EC was cultured with other types of neurovascular cells,^51^ such as pericytes and astrocytes, due to their supply of promoting factors for the BBB formation (Figure 2C,D).^25,42^ Also, previous work where TEER was monitored over the days of culture, showed significance difference in values due to the optimal incubation time for EC to develop TJs properly.^21,52^ Moreover, several studies demonstrated that shear stress has a mechanotransductive effect that up-regulates the TJs expressions and RNA levels of BBB transporters.^53,54^ As well, most BBB-oCs with applied shear stress showed higher electrical resistance (Figure 2E–H).^22,40,42,45^ Remarkably, it is important to consider the use of in vivo brain microcapillaries shear force values (5–25 dyn/cm^2^)^48,55,56^ to mimic a more reliable BBB environment. However, until now authors have predominantly used shear forces below in vivo values, except some who applied a physiological shear stress about 5.8–20 dyn/cm^2^.^11,35,45,57,58^ One of the main reasons why physiological shear cannot be applied is the limitation to reduce the dimension of the BBB-oC channel, which is inversely related to shear stress.

## Future Oportunities for the Integration of Biosensors in BBB-oC

TEER values could be influenced by multiple factors and demonstrate selectivity issues. Biosensors permits one to increase selectivity and to detect a wide range of analytes to further expand the applications of BBB-oC by including the detection of disease-specific analytes for a deeper understanding on NDDs progression and drug permeation and testing its performance in brain-based cells. Despite the great advantages offered by this technology, biosensors integrated in BBB-oC have not yet been reported. This section proposes the most relevant biomarkers to monitor the main NDDs related with damage to the BBB, as well as analytes to evaluate drug cytotoxicity (Table 1). The possible designs and configurations of these sensors are also described, paying special attention to automatized analysis for the monitoring of disease evolution on the chip to take BBB-oC technology a step further

**Table 1:** Proposed Analytes and Biosensors for BBB-oC Monitoring

detection focus	analytes/biomarkers	recommended bipreceptor	recommended biosensor
drugs	Paclitaxel, Simvastatine, Fluvastatin, ...	aptamer	Aptabeacon
cytotoxicity	LDH and/or glutamate	lactate oxidase and glutamate oxidase	enzymatic sensor
ions	Ca^+^2, Na^+^, K^+^, and/or Fe^2+/3+^	ionophores	ISE
neuro-inflammation markers	cytokines, chemokines, CAMS, MMPs	aptamer and/or antibody	aptabeacon and/or impedance immunosensor
ROS	hydrogen peroxide	HRP	enzymatic sensor

**Figure 3:**
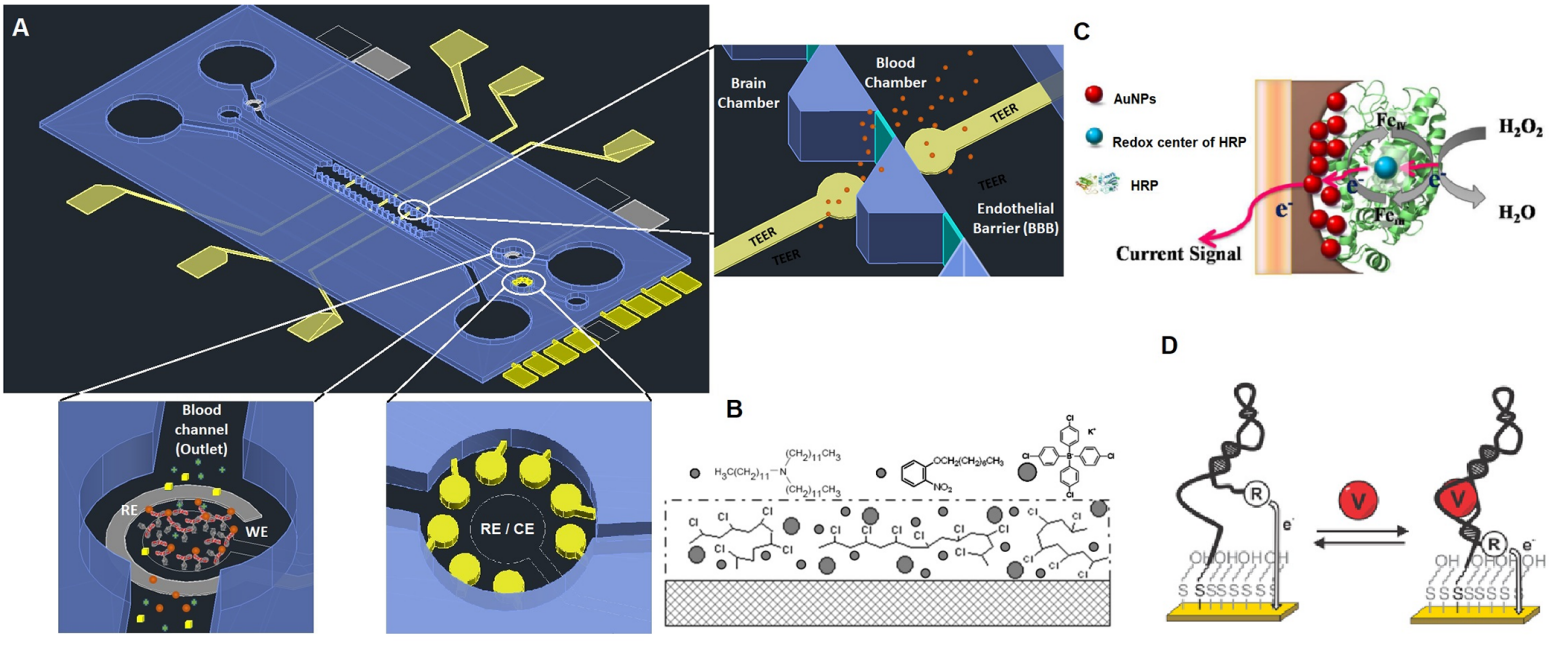
(A) Flank design of a BBB-oC considering two blood channels with a central brain chamber. Detail of channels, where biosensors or array of sensors has been integrated, and TEER electrodes on each side of the endothelial barrier. (B) Schematic drawing of ion-selective membrane for potassium detection. Reproduced with permission from ref (61). Copyright 2014 The Authors under Creative Commons Attribution 3.0 Unported License, published by MCPI. (C) Illustration of the mechanism underlying the detection of H_2_O_2_ with HRP-AuNPs. Reproduced with permission of ref (62). Copyright 2015 The Authors under Creative Commons Attribution 4.0 International License, published by PLOS. (D) Scheme of the aptabecon for drugs binding-induced change in the electron. Reproduced with permission from ref (63). Copyright 2019 American Chemical Society.

In biosensors, optical detection is one of the most widely used methods to read out, such as surface plasmon resonance and optical waveguide spectroscopy where the physical behavior of the sensor surface is changing upon the interaction with the analyte. Also, fluorescence and colorimetric labeled antibodies are used in a sandwich format to elucidate the interaction with the analyte. However, to integrate real-time continuous monitoring into OoC platforms, the transducer must be miniaturized at low cost and integrated into microfluidic channels with compatible fabrication techniques (Figure 3A). Also, it is desirable that the sensor be label-free and reagent-free for a continuous monitoring, which is not always possible in optics read-out systems. In this direction, electrochemical transducers are attractive because the recorded signal of the electrochemical reaction does not need translation into a digital signal, the reading equipment being less expensive and their microfabrication technology affordable for electrochemical sensors integration in microfluidics.^59,60^

### Drugs Analysis

Currently, the most extended use for the BBB-oC platforms is the study of drug delivery to the brain through the BBB. The usual method to characterize the drug transport across the brain barrier is by quantification with external analysis of the remaining drug in the blood channel, using reverse-phase high-performance liquid chromatography (HPLC).^64^ But this technology is not available in most laboratories because it is expensive and requires a specialized analytical chemist. An integrated specific sensor for the drug may allow the automatization of the process at lower cost. Most of the drugs used in NDDs are low molecular weight (∼150–300 Da) organic molecules. Then, the most efficient bioreceptor in biosensors for these types of analytes are aptamers, which are a synthetic DNA strain able to fold into well-defined three-dimensional structures and able to bind with a corresponding target through molecular recognition.^65,66^ An electrochemical aptamer-beacon-based sensor is an excellent candidate for label-free and reagent-free analyses for integrated in situ detection.^67,68^ The binding of the analyte with the redox labeled aptamer induces a 3D conformational change with a subsequent modification on the distance of the redox tag with respect to the electrode modifying the electron transfer kinetics of the redox tag (Figure 3A). Electrochemical aptabeacon has been reported for drug monitoring (vancomycin) in plasma using gold electrodes and a self-assembled monolayer of thiolated-aptamer methylene blue labeled (Figure 3D).^63^

### Cytotoxicity Monitoring

Besides the monitoring of drug permeability across the BBB, it is also relevant to analyze the toxicity generated in the brain vasculature by the medication. Cytotoxicity of drugs can be monitored by the detection of secreted markers in the extracellular matrix of the cell culture. Some authors evaluated the cytotoxicity of ECs on the basis of lactate dehydrogenase (LDH) by colorimetric quantification or fluorescence immune staining of the death cells,^69^ but both techniques do not measure in real time, detecting late apoptosis and requiring culture fixation. Integrated biosensors would offer real-time monitoring and quantification of early apoptosis in an automatized way. For this specific analysis an electrochemical enzymatic biosensor for LDH detection can be used. Enzymatic sensors are based on the very specific lock and key model between the enzyme and its substrate, producing a catalytic reaction with exponential product formation. Most of the enzymatic interactions are associated with a reduction–oxidation reaction that produces electrons, electrochemical detection being the best candidate. Therefore, enzymes could be an attractive bioreceptor to determine by electrochemistry certain molecules in NDDs, permitting low limits of detection, high selectivity, label-free sensing, and reusability due to reversible enzyme–substrate interaction. The integration of an enzymatic sensor in OoC proceeds by the immobilization on the electrode of the enzyme associated with a redox mediator, which helps in the electron transfer of the produced electrodes in the redox reaction to the electrode to perform amperometric detection. For LDH detection, an electrochemical enzymatic sensor functionalized with lactate oxidase and a redox mediator can be used, such as that reported by Park et al.^70^ Another real-time biomarker for cell cytotoxicity analysis is glutamate that can be affected by neuronal toxicity. Also, in this case electrochemical enzymatic sensors based on glutamate oxidase or glutamate dehydrogenase are excellent candidates for this purpose.^71,72^

To get the complete picture of an in vitro model of NDD where the neurovasculature is affected and to analyze the neuronal toxicity of drugs permeated through the BBB, it is required to combine the coculture of cells from the cerebral vasculature with neurons. The progressive degeneration of the activity of the neurons offers a clear evolution of the NDD, being relevant to detect it from an early stage. The physiological function of neurons is to send information to neighboring neurons over a long distance in the form of electrical impulses, which involves ions flow (sodium, potassium, calcium, and chlorine) through ion channels. The degradation of neurons causes neuronal activity to be reduced, affecting the flow of ions. Therefore, the change in the concentration of these ions in the extracellular matrix is an indicator of the neuron’s dysfunction. Ion-selective electrodes (ISE) are based on organic molecules (ionophores or ion-exchange substance) contained in a polymeric matrix as poly(vinyl chloride) drop cast on the working electrode. Here, the ions are attracted by the ionophore and the attachment of the ions on the working electrode generates a potential difference with respect to the reference electrode (Figure 3B). This difference can be measured by potentiometry in a reagent-less and label-free manner.^73^ The most typical chemical receptors in sensors for the neuronal involved ions are valinomycin for potassium detection,^74^ bis[(12-crown-4)methyl] 2,2-didodecylmalonate for sodium detection,^75^ and bis(2-ethylhexyl) adipate for calcium detection.^70^ However, Na^+^, K^+^, and Ca^2+^ monitoring in cell culture has some difficulties due to the presence of these ions in the cell medium and so poor signal-to-noise ratio as well as a fast change on the ion’s concentrations, which limit its usefulness for analyzing neuronal activity. Even so, some examples of neural analysis with ISE can be found in the literature, such as the microelectrode modified with tetraphenylarsonium tetrakis(*p*-biphenylyl)borate contained in poly(vinyl chloride) membrane for calcium detection in the intracellular matrix of giant neurons.^76^

### Inflammation and Oxidative Stress Biomarkers

One of the most important pathological hallmarks of many NDDs contributing to cognitive decline is the increase of the BBB permeability.^77^ Vascular damage in NNDs is produced mainly by neuroinflammation, caused by either pathogens or trauma, and involves both the vascular and immune systems.^78^ Secreted inflammatory cytokines, such as IL-1β, IL-6, IL-17, IFN-γ, and TNF-α, regulate the expression and configuration of a cell junction’s proteins in the ECs of the BBB, altering the barrier permeability.^79,80^ Moreover, these cytokines up-regulate, individually or synergistically, the expression of pro-inflammatory chemokines, such as CCL2, CCL3, CCL4, CCL5, CXCL8, CXCL9, CXCL10, and CX3CL1,^81,82^ and cell-adhesion molecules (CAMs), such as ICAM-1, VCAM-1, ALCAM, MCAM, E-selectin, and P-selectin in the ECs of the BBB.^83,84^ These chemokines and CAMs promote leukocytes adhesion to ECs and facilitate their extravasation across the BBB aided by matrix metalloproteinases (MMPs).^85,86^ Several MMPs such as ICAM-1, VCAM-1, ALCAM, MCAM, E-selectin, and P-selectin, which are critical in tissue remodeling, are also involved in the neuroinflammation process either acting as signaling molecules in neuroinflammatory pathways or proteolyzing cerebrovascular basement membrane and TJ proteins.^87^ Leukocyte infiltration across the BBB initiates a series of events mostly leading to demyelination and axonal loss, thereby causing severe neuronal damage.^88^ During this process, there is an active production of reactive oxygen species (ROS) from activated microglia and macrophages.^89,90^ The superoxide anion (O_2_^–^), one of the most abundant of ROS, can easily react with nitric oxide to generate peroxynitrite anion (ONOO^–^), a powerful oxidant for proteins that alters their normal function.^91^ These include TJs proteins and signaling proteins, which can be promoted again an inflammatory response. Thus, oxidative stress and inflammation are involved in a kind of self-perpetuating cycle,^88^ producing multiple biomarkers that warn about the different stages of this process, which are very interesting to analyze in the in vitro model to understand the evolution of the disease on the chip.

Neuroinflammation is a known protective mechanism of the brain, and it is also a common characteristic in the pathogenesis of several neurodegenerative diseases such as AD, PD, ALS, and multiple sclerosis (MS), among others.^92^ Therefore, the biomarkers of neuroinflammation, which directly affect the BBB permeability, can be considered as nonspecific markers of the aforementioned NDDs, being useful for the study of different diseases models. Most of the neuroinflammatory biomarkers specified (cytokines, chemokines, CAMs, and MMPs) are proteins.

Biosensors can be integrated in BBB-oC devices, placed in the outlet of the brain chamber of the OoC, for real-time monitoring of these biomarkers using aptamers and/or antibodies for sensing proteins as previously discussed. For the detection of this interaction, a labeling through a secondary labeled antibody in a sandwich format is usually required in the sensor. However, for continuous and real-time monitoring is required a label-free and reagent-less biosensor. Thus, an attractive option to include in the OoC is an impedance spectroscopy electrochemical read out, which has high sensitivity without requiring labeling.^38^ This technique permits one to monitor the evolution of the electrical circuit created on the sensor surface, which is modified by the interaction between the antigen–antibody complex. Examples of impedimetric immunosensor for diagnosis purpose has been reported for the neuroinflammatory biomarkers; cytokines,^93^ chemokines,^94^ CAMs,^95^ and MMPs,^96^ as well as aptabeacons for IFN-γ cytokine analysis by Liu et al.^97^

Another important factor in detecting neuroinflammation is monitoring ROS production. Hydrogen peroxide (H_2_O_2_) is the most stable ROS and, therefore, one of the preferred targets for ROS analysis. Different enzymes such as HRP,^62^ and superoxide dismutase^98^ have been used in the detection of ROS, more common being the use of HRP enzyme as bioreceptor (Figure 3C). For ROS monitoring in a BBB-oC, screen-printed, or inkjet-printed carbon electrodes combined with an Ag/AgCl or Pt (more expensive but less cytotoxic) reference electrode can be integrated in the microfluidic outlet channel of the brain coculture chamber. The carbon electrode needs to be functionalized with HRP enzyme combined with redox mediator for the amperometric detection of H_2_O_2_.^99^

The NDDs share common hallmarks, which bring many advantages in the technology development since one platform elaborated for a specific analyte can be useful for the study or application in different NDDs in OoC.

## Conclusions and Future Outlook

The OoC gives us a closer view of the specific parts and minimal functions of an organ allowing a detailed simulation and the study of the mechanical and physiological responses related to different pathologies. Although great progress has been made in the field of BBB-oC, these platforms are still at a very early stage as the developed systems are a very simplified adaptation of the real physiology of the BBB. Currently, the cell culture development into the BBB-oC is widely monitored by fluorescence labeling of specific proteins with a confocal microscope. This method is time-consuming and costly and does not permit a real-time monitoring and automatization of the detection, hindering the access of this technology to the market. Currently, TEER is the only type of integrated sensor in BBB-oC that contains all of these advantages. The wide range of electrode materials, shapes, sizes, and configurations for TEER measurement was described in this review, advising the most efficient configuration. However, this type of measurement brings limited information about the BBB and suffers from the influence of many variables (temperature, electrolyte concentration, and others) in the response leading to conflicting results. With the commented-on difficulties, examples of BBB-oC have been commercialized. Besides the generic barrier integrity, new BBB-oC models are expected to go one step further and allow for the continuous monitoring of markers of inflammation and cell damage. Therefore, the integration of biosensor’s technology opens many possibilities for sensing on this type of device for continuous monitoring of many different analytes. The continuous control of the concentration evolution of various molecules inside the cell culture of the BBB-oC may contribute with relevant information for a deeper knowledge of the disease correlated with BBB permeability and drug testing. Moreover, the pathological similarities between NDDs simplify the technology development since the same biosensor can be useful for the study of different illnesses. The disruption of the BBB is a consequence in various NDDs, and as mentioned before there are many different biomarkers evolving that need a closer study. This Perspective brings a forward-looking vision in the BBB-oC area to develop multiple fully automated selective analyzers, describing the most relevant biomarkers in NDDs correlated with the BBB and the possible biosensors strategies that could be performed to be integrated into BBB-oC to measure and monitor these molecules.

As we have already advanced in the Perspective, this technology should move in the direction of a fully automated multianalyzer to be used in a wider range of applications. For this technology to reach a wider market, the BBB-oC must be designed as an easy-to-use analyzer tool to be used for drug testing or custom drugs in NDD in the hospital or pharmaceutical industry. We hope that this work will help to develop more accurate and reliable TEER measurements in the future BBB-oC and allow the availability of new representative BBB models integrating biosensors for the study and real-time detection of new analytes in the chip.
